# Comparison of the Oxford COVID‐19 Government Response Tracker and the ECDC‐JRC Response Measures Database for nonpharmaceutical interventions

**DOI:** 10.1111/irv.13249

**Published:** 2024-01-02

**Authors:** Susanne Heemskerk, Peter Spreeuwenberg, Harish Nair, John Paget, PROMISE investigators

**Affiliations:** ^1^ Netherlands Institute for Health Services Research (Nivel) Utrecht the Netherlands; ^2^ Centre for Global Health, Usher Institute University of Edinburgh Edinburgh; ^3^ School of Public Health University of the Witwatersrand Johannesburg South Africa

**Keywords:** COVID‐19, disease outbreaks, epidemiology, non‐pharmaceutical interventions, public health

To the Editor,

During the COVID‐19 pandemic, governments implemented different public health measures and interventions to control COVID‐19. These wide‐ranging public health interventions, also known as non‐pharmaceutical interventions (NPIs), have been documented in Europe in two different publicly available databases. In the context of an EU‐funded research project aimed at Preparing for Respiratory Syncytial Virus (RSV) Immunisation and Surveillance in Europe (PROMISE),^1^ we will use these databases to assess the impact of NPIs (e.g., school closures) on the seasonality of RSV. In this letter, we will focus on the comparison of the NPI databases, which we will later use for our analyses.

The first database is the Oxford COVID‐19 Government Response Tracker (OxCGRT)^2^ which collects policy measures implemented from 01 January 2020 to 31 December 2022 in 185 countries and contains 25 indicators that are recorded on an ordinal scale that represents the level of strictness of the policy. The second database is the European Centre for Disease Prevention and Control (ECDC) and the Joint Research Centre (JRC) Response Measures Database (ECDC‐JRC RMD)^3^ which is an archive of NPIs introduced by 30 countries in the EU and EEA from 01 January 2020 to 30 September 2022.

We compared five NPI measures related to RSV transmission in the OxCGRT and ECDC‐JRC RMD databases and found important differences (see Figure 1). We chose five measures with similar definitions: workplace measures, public gathering restrictions, closure of public spaces, closure of educational institutions and protective mask use. We chose the measures that were most comparable across both databases and chose the strictest measure to define whether an intervention was implemented or not (e.g., we chose full implementation over partial implementation to assess whether a measure was introduced).

**FIGURE 1:**
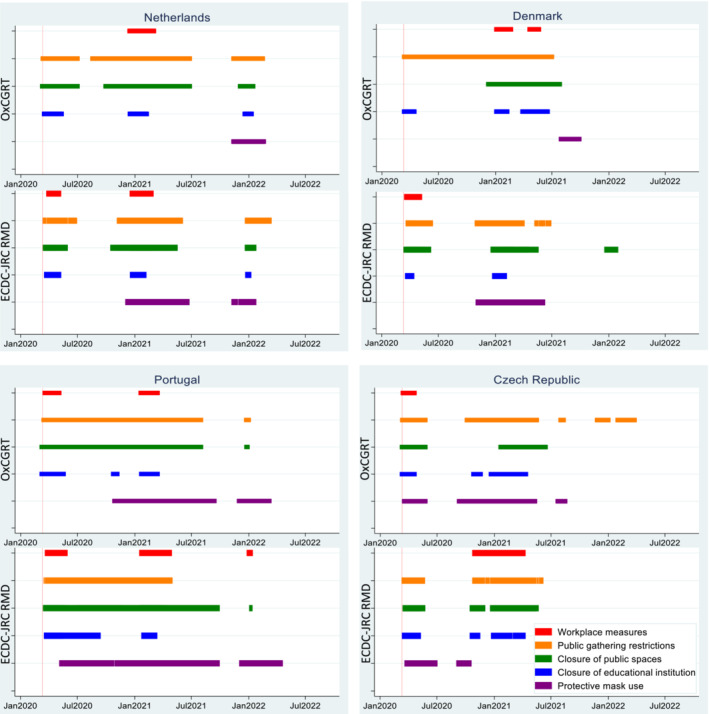
Comparison of the Oxford COVID‐19 Government Response Tracker (OxCGRT) and ECDC‐JRC Response Measures Database (ECDC‐JRC RMD) in four countries. The following measures were considered: workplace measures include closing (or work from home) for all‐but‐essential workplaces (OxCGRT) and closure of workplaces and teleworking (ECDC‐JRC RMD). Public gathering restrictions include limits on gatherings between 0 and 100 people (OxCGRT) and limits participation of indoor/outdoor attendance between 31 and 100 people and ban on all events (ECDC‐JRC RMD). Closure of public spaces include cancelling of public events (OxCGRT) and public spaces, for example, entertainment venues, sport centres, hotels and nonessential shops (ECDC‐JRC RMD). Closure of educational institutions include closing of schools and universities* (OxCGRT) and daycare nursery and primary schools (ECDC‐JRC RMD). Protective mask use include requirement of facial coverings outside the home with people present and at all times regardless of location and presence of other people (OxCGRT) and protective mask use in closed and public spaces (ECDC‐JRC RMD). The red line indicates 11 March 2020, when the World Health Organization declared COVID‐19 a pandemic.^4^ *childcare and nurseries do not count as educational closures for OxCGRT.

The four countries in Figure 1 were selected to capture different regions in Europe, ranging from Portugal in the West to the Czech Republic in the East and Denmark and the Netherlands in Northern Europe. The figure shows that the measures in the two databases are often similar, but differences between the start and end dates for each NPI are clearly observed. For example, in Denmark, public gathering restrictions started in March 2020 and ended around August 2021 according to the OxCGRT, while the ECDC‐JRC RMD shows approximately the same start and end date but with multiple weeks where public gatherings restrictions were not applied. It is difficult to observe clear patterns in the differences between the two databases, and it is not possible to say which database is more conservative or strict (i.e., one database consistently indicates shorter intervention periods).

Another study compared international border restrictions in four countries (Morocco, New Zealand, South Korea and the United States) across five NPI databases, including OxCGRT and also found discrepancies between the timing of interventions.^5^ The variation between the databases might be explained by differences in definitions, the methodology regarding how the databases are constructed or the way the data were collected. It is also possible that one database is better for certain indicators, while the other is better for other indicators. Considering these points, it is not possible for us to say which NPI database is best and it may be advisable for researchers to run their analyses on both databases (separately).

Our assessment finds that the OxCGRT and ECDC‐JRC RMD databases are valuable for research purposes; they are comprehensive, freely available and easily accessible. However, despite the extensive documentation provided, we encountered challenges in synchronising the databases and we observed many disparities. This means it is difficult for researchers to select a suitable NPI database for research purposes, including for our modelling study. In summary, we found important differences between the two databases regarding NPIs related to RSV and we would recommend that an evaluation of the databases (e.g., accuracy and completeness) is initiated to support other researchers wanting to use these databases for research purposes.

## AUTHOR CONTRIBUTIONS

SH and JP have contributed to the conception and design of the study. SH was responsible for data analysis and interpretation of the data. SH wrote the letter, and JP revised all versions. JP was involved until one of the final versions of the letter; after his passing, only small textual changes occurred, with no substantive alterations taking place. PS and HN critically reviewed the manuscript, provided comments and approved this manuscript.

## CONFLICT OF INTEREST STATEMENT

JP declares that Nivel has received unrestricted grants from the World Health Organization, Sanofi and the Foundation for Influenza Epidemiology outside the submitted work. HN reports grants from the World Health Organization, the National Institute for Health Research, Pfizer and Icosavax and personal fees from the Bill & Melinda Gates Foundation, Pfizer, GSK, Merck, AbbVie, Janssen, Icosavax, Sanofi, Novavax, outside the submitted work.

### PEER REVIEW

The peer review history for this article is available at https://www.webofscience.com/api/gateway/wos/peer-review/10.1111/irv.13249.

## Data Availability

All data are presented in the letter.
